# Editorial: Inter-cultural cooperation: The role of attitudes, (shared) expectations, and behavioral standards

**DOI:** 10.3389/fpsyg.2022.1044588

**Published:** 2022-11-22

**Authors:** Gari Walkowitz, Alexis Belianin, Angela Rachael Dorrough

**Affiliations:** ^1^Freiberg University of Mining and Technology, Freiberg, Germany; ^2^Technical University of Munich, Munich, Germany; ^3^National Research University Higher School of Economics, Moscow, Russia; ^4^Department of Social Psychology, University of Cologne, Cologne, Germany

**Keywords:** inter-cultural psychology, prosocial behavior, cooperation, cross-cultural measure invariance, refugees and asylum seekers, migration

Social interactions transcending national contexts and individuals' cultural imprints are omnipresent in the contemporary world. This includes, but is not limited to, personal encounters, multi-ethnic teams in organizations, and not least, political conflicts. Beyond global business activities, developments of the last few years have added new dimensions to social cooperation and conflict across national borders. For example, fostered increase in digitized communication due to pandemic restrictions (Carroll and Conboy, 2020) has contributed to a rise in global inter-personal interactions, whose implications are yet to be explored. In addition, military conflicts of the XXI century, besides direct casualties and damage, are likely to cause multiple waves of mistrust, hostilities, and national displacements of millions of people who depend on interacting and cooperating with the local populations in the receiving countries.

The goal of the Research Topic is to offer new perspectives for reflecting and disentangling the determining factors of inter-cultural cooperation. A distinction is made between an intra-cultural and an inter-cultural view to better understand the potential impact of attitudes formed within national cultures on behavior in inter-cultural situations. The collected papers cover and address different forms of intra- and inter-cultural social interactions, especially pro-social behaviors, in a variety of ways. Some of these contribute to our knowledge and understanding of important phenomena of the contemporary world, such as attitudes toward migrants, or experiences of refugees. More generally, all papers make scientific contributions by providing grounded evidence on interconnections between individual values, experiences, preferences, and behavior. No less importantly, these works also yield several null findings, or the absence of expected causalities, thus enhancing our knowledge of the potential limits of existing theories and concepts connecting them.

In their contribution, *The Power of Tolerance vs. Unselfishness as a Cultural Determinant of Cooperation*, Eriksson et al. address the impact of stressing particular societal values on behavior in intra-cultural and inter-cultural social dilemmas. By re-analyzing and comparing existing large-scale multinational data sets from 62 societies (from Eriksson et al., 2021), the authors show that societies with a high cooperation level—as measured by money transfers in prisoner's dilemma games—exhibit stronger cooperation norms. Behavior in a country, as well as behavioral norms, also predict meta-norms concerning how to act against uncooperative individuals. This work further addresses the question of whether cultural values (i.e., “unselfishness” and “tolerance”) are associated with cooperative behaviors and cooperative norms, respectively. The authors argue that tolerance might be a good predictor of trust, especially in modern global and heterogeneous societies. Using correlation and multilevel analyses, they show that cooperation behavior and norms seem to be independent of how much a society values unselfishness. However, emphasis on tolerance and respect for other people is a good predictor for cooperative behaviors and the related (meta-)norms, i.e., the treatment of non-cooperators. These connections are found to be especially pronounced in inter-cultural cooperation, which supports the mediating role of tolerance in sustaining cooperation across national borders.

Wang et al. investigate behavioral differences from an intra-cultural and culture-comparative perspective in their paper *Differences in Mood, Optimism, and Risk-Taking Behavior Between American and Chinese College Students*. Cooperation often requires an element of trust, and the inclination to trust may in turn depend on how much a person is willing to take risks (e.g., Bohnet and Zeckhauser, 2004; Eckel and Wilson, 2004; Lönnqvist et al., 2015). The paper's findings underscore the fact that well-established findings—typically derived from Western participant pools—can differ between countries and that one should be careful about the generalizability of psychological findings. The authors show that a more positive mood and optimism may influence American participants to take more risks and may influence Chinese participants to take fewer risks. Mood interacts with optimism in influencing risk-taking in both the American and Chinese samples but in different ways. Specifically, the association between more optimism and more risk-taking in American students is weaker among those with a more positive mood, while for the Chinese, higher optimism and positive mood correlate with less risk-taking. The authors attribute the opposite effects to the collectivistic nature of the Chinese culture which puts more pressure on the need to succeed in front of one's peers.

Caused by the globalization of economic activity and the increase in social and political migration, the frequency of inter-cultural contacts went up in many countries in recent years. Is it reasonable to assume that so did people's abilities to effectively communicate with members from different nations? Have people developed their inter-cultural competences (Deardorff, 2006) and their cultural intelligence (Ang and Van Dyne, 2008) accordingly? The third paper *Scale Characteristics of Intercultural Competence Measures and the Effects of Intercultural Competence on Prejudice* by Genkova et al. studies this connection using the cultural intelligence scale (Van Dyne et al., 2008), the multi-cultural personality questionnaire (Van der Zee et al., 2013), as well as a blatant and subtle prejudice scale (Pettigrew and Meertens, 1995). In contrast to previous research, the authors sample Eastern European countries (Czech Republic, Hungary, and Serbia) typically underrepresented in psychological research and contrast their response scores with data obtained in a Western European country (Germany). While cultural intelligence is mostly found to be negatively associated with prejudice indices, the effects of cultural personality characteristics and cultural competence as mediators are different in the different samples and inconclusive. Besides, this paper makes a noteworthy methodological contribution by studying whether different scales used in a multi-cultural context exhibit different factor structures in different countries. For all applied scales, the authors find violations of measurement invariance highlighting the potentially limited generalizability of findings across WEIRD and non-WEIRD countries (e.g., Henrich et al., 2010) and thus the importance of adapting well-established scales to different cultural contexts.

Finally, two papers study inter-cultural encounters within the highly topical subject of migration focusing on pro-social behavior and attitudes toward refugees. In the work *Altruistic Giving Toward Refugees: Identifying Factors That Increase Citizens' Willingness to Help* by Hellmann et al. German student participants played dictator games where the identity of the receivers was systematically varied: student or non-student, member of own local community (Bonn), or another community (Cologne), or a refugee who, in turn, can also be local or non-local. The authors' main finding is the (hypothesized) prevalence of ingroup bias (e.g., Jackson and Esses, 2000) in altruism toward ingroup members from the same city spreading over to local refugees. Specifically, participants transferred more resources to refugees from the same city compared to another city. Results also reveal that participants with a left-wing and a pro-social orientation are more inclined to support refugees. The findings underline the importance of local identity in inter-cultural and political contexts for understanding the inclination to cooperate with strangers even if these identities may have risen in the short-term or by chance.

The paper *Trauma and Trust: How War Exposure Shapes Social and Institutional Trust among Refugees*, written by Hall and Werner, presents data from a field study collected in a refugee camp in Southern Turkey. Participants—over 800 long-term refugees from Syria and Iraq—answered a comprehensive questionnaire on individual exposure to armed conflicts (Arnetz et al., 2014), symptoms of posttraumatic stress (Lang and Stein, 2005), and posttraumatic growth (Cann et al., 2010). The authors investigate how individual differences in these indicators shape two types of trust among refugees: generalized social trust and trust in the institutions of the settlement country. They find that trust in local institutions of the host country (Turkey)—in particular, courts and police—is higher for those who have been exposed to military actions and subjected to personal violence and emotionally powerful stimuli. Respondents who have been directly exposed to violence may increase their institutional and social trust in reciprocation toward those instances who have helped them to escape from adverse conditions—but only if those experiences have been personal (to oneself or a close person) and direct.

The topic of inter- and intra-cultural cooperation remains broad and growing in its scope and importance if we consider the number of instances when it potentially matters and the growing intensity of inter-cultural conflict in the contemporary world. The papers presented in this Research Topic can, therefore, only reflect an excerpt of research on inter-cultural social interactions. Nevertheless, the contributions identify important predictors of inter-cultural pro-sociality and cooperation such as society's valuation of tolerance and respect for other people, the influence of personal mood and optimism on the propensity to take risks, common local identity, own exposure to violence, and received support by local authorities. Together with the methodological insight that the suitability of established measurement tools needs to be critically evaluated within inter-cultural and non-Western research contexts, this body of evidence can help to design interventions aiming at facilitating social interactions across national borders, and to broaden our knowledge of the manifold facets of inter-cultural cooperation and conflicts.

## Author contributions

All authors listed have made a substantial, direct, and intellectual contribution to the work and approved it for publication.

## Funding

GW and AB prepared the article in the framework of a research grant funded by the Ministry of Science and Higher Education of the Russian Federation (Grant ID: 075-15-2022-325). This work was supported by the Zentrum Digitalisierung.Bayern (ZD.B).

## Conflict of interest

The authors declare that the research was conducted in the absence of any commercial or financial relationships that could be construed as a potential conflict of interest.

## Publisher's note

All claims expressed in this article are solely those of the authors and do not necessarily represent those of their affiliated organizations, or those of the publisher, the editors and the reviewers. Any product that may be evaluated in this article, or claim that may be made by its manufacturer, is not guaranteed or endorsed by the publisher.
